# Grave Situation in the Liverpool District

**Published:** 1911-08-19

**Authors:** 


### THE STRIKE AND THE HOSPITALS.

### GRAVE SITUATION IN THE LIVERPOOL DISTRICT.

As we go to press, the very serious danger caused by the spread of the strike, has led the hospitals of Liverpool, which at present is the centre of the labour trouble, to send their representatives to a special meeting which was held on the 15th instant. A large number of representatives attended, and the result has been the dispatch of a warning to the Lord Mayor and to the Strike Committee, to the effect that the hospitals will be closed unless their provisions are allowed to go through. The fact that actual bloodshed has occurred already will make the gravity of this warning on the part of the hospitals apparent. The attitude taken by the hospitals seems to us the most statesmanlike course that is open to a body which has no executive powers. It should certainly help the cause of order.

### THE STRIKE AND THE LIVERPOOL ROYAL INFIRMARY.

On the whole, our inquiries at the various hospitals of the country show that the strike committees at first did everything that they could to facilitate the reception of the necessary stores of food and ice, from the interruption of which many other sections of the community have suffered. At the commencement of the strike in Liverpool, the Royal Infirmary there had its milk-supply held up till the Strike Committee became aware of what was happening, since when, by its courtesy, there was, we understand, no further trouble till the early part of this week, when the Infirmary has been faced with the possibility of closing its wards from the difficulty of obtaining necessities. The medical officers, we learn on going to press, have been tentatively instructed not to admit cases from outside districts. During the riot last Sunday the out-patient department at the Infirmary was not unlike a small shambles; over a hundred cases had to be attended to within an hour. The local contractors are keeping the Royal Infirmary regularly supplied with everything except fresh butter, which is hard to secure. Cotton dressings, which came from Manchester by passenger train, had to be sent for, and some hardware articles for the new out-patient department were still on the railways on Monday last. At Ipswich the East Suffolk and Ipswich Hospital only suffered one day from the late arrival of its fish supply.

### LONDON HOSPITALS AND THE STRIKE.

In London, the Seamen's Hospital, Greenwich, failed only to secure fresh butter, and oxygen had to be sent for last week, the usual channel for receiving it failing to deliver. At the London Fever Hospital the fish was not delivered on Friday, the 11th, and on one occasion the ice cart was prevented from approaching the gates till, like the provision cart which was marked "hospital supplies," it was allowed to go through. On Friday last week, in short, no ice and no fish were obtainable at this institution. The Prince of Wales' General Hospital, Tottenham, came off well, as all its provisions are obtained locally, even ice, which has been supplied regularly by the Imperial Cold Stores. The London Hospital, which has a reputation for meeting every emergency, had no difficulty, for the simple reason that it has an ice plant of its own. The contractors also had no difficulty in delivering goods as soon as it was known that they were for the London. In short, it has been the children and the sick of the strikers themselves who, in spite of some lay papers, have suffered apparently more than most people.

